# Comparative study of the mycorrhizal root transcriptomes of wild and cultivated rice in response to the pathogen *Magnaporthe oryzae*

**DOI:** 10.1186/s12284-019-0287-9

**Published:** 2019-05-10

**Authors:** Lei Tian, Chunling Chang, Lina Ma, Fahad Nasir, Jianfeng Zhang, Weiqiang Li, Lam-Son Phan Tran, Chunjie Tian

**Affiliations:** 1 Key Laboratory of Mollisols Agroecology, Northeast Institute of Geography and Agroecology, Chinese Academy of Sciences, Changchun, 130102 China; 2 University of Chinese Academy of Sciences, Beijing, 100049 China; 3 School of Life Sciences, Northeast Normal University, Changchun City, Jilin China; 4 College of Life Science, Jilin Agricultural University, Changchun, Jilin China; 5 Stress Adaptation Research Unit, RIKEN Center for Sustainable Resource Science, 1-7-22, Suehiro-cho, Tsurumi, Yokohama, 230-0045 Japan; 6 Institute of Research and Development, Duy Tan University, 03 Quang Trung, Da Nang, 550000 Vietnam

**Keywords:** Arbuscular mycorrhizal fungi, Cultivated rice, *Magnaporthe oryzae*, Transcriptome analysis, Wild rice

## Abstract

**Background:**

Rice, which serves as a staple food for more than half of the world’s population, is very susceptible to the pathogenic fungus, *Magnaporthe oryzae*. However, common wild rice (*Oryza rufipogon*), which is the ancestor of Asian cultivated rice (*O. sativa*), has significant potential as a genetic source of resistance to *M. oryzae*. Recent studies have shown that the domestication of rice has altered its relationship to symbiotic arbuscular mycorrhizae. A comparative response of wild and domestic rice inhabited by mycorrhizae to infection by *M. oryzae* has not been documented.

**Results:**

In the current study, roots of wild and cultivated rice colonized with the arbuscular mycorrhizal (AM) fungus (AMF) *Rhizoglomus intraradices* were used to compare the transcriptomic responses of the two species to infection by *M. oryzae*. Phenotypic analysis indicated that the colonization of wild and cultivated rice with *R. intraradices* improved the resistance of both genotypes to *M. oryzae*. Wild AM rice, however, was more resistant to *M. oryzae* than the cultivated AM rice, as well as nonmycorrhizal roots of wild rice. Transcriptome analysis indicated that the mechanisms regulating the responses of wild and cultivated AM rice to *M. oryzae* invasion were significantly different. The expression of a greater number of genes was changed in wild AM rice than in cultivated AM rice in response to the pathogen. Both wild and cultivated AM rice exhibited a shared response to *M. oryzae* which included genes related to the auxin and salicylic acid pathways; all of these play important roles in pathogenesis-related protein synthesis. In wild AM rice, secondary metabolic and biotic stress-related analyses indicated that the jasmonic acid synthesis-related α-linolenic acid pathway, the phenolic and terpenoid pathways, as well as the phenolic and terpenoid syntheses-related mevalonate (MVA) pathway were more affected by the pathogen. Genes related to these pathways were more significantly enriched in wild AM rice than in cultivated AM rice in response to *M. oryzae*. On the other hand, genes associated with the ‘brassinosteroid biosynthesis’ were more enriched in cultivated AM rice.

**Conclusions:**

The AMF *R. intraradices-*colonized rice plants exhibited greater resistance to *M. oryzae* than non-AMF-colonized plants*.* The findings of the current study demonstrate the potential effects of crop domestication on the benefits received by the host via root colonization with AMF(s), and provide new information on the underlying molecular mechanisms. In addition, results of this study can also help develop guidelines for the applications of AMF(s) when planting rice.

**Electronic supplementary material:**

The online version of this article (10.1186/s12284-019-0287-9) contains supplementary material, which is available to authorized users.

## Background

Approximately two-thirds of the current human population subsist on rice (*Oryza sativa*) as their staple food (Talukdar et al. 2017). However, as the human population continues to increase, and land for planting rice decreases, the need to increase rice production is critical. Pathogenic fungi are one of the limiting factors for rice production. *Magnaporthe oryzae*, a pathogenic fungus that is the causal agent of rice blast disease, is widely distributed and causes serious reductions in rice yields worldwide (Liu et al. 2010; Wang et al. 2017). The breeding approach to create disease-resistant rice varieties is commonly used to improve rice production. Common wild rice (*O. rufipogon*), the source for the breeding of cultivated rice, and a relative of Asian cultivated rice, represents sources for several desirable attributes, including disease resistance (Hua et al. 2015; Mao et al. 2015; Liang et al. 2017). For example, Zhang et al. (1998) identified a new gene in common wild rice involved in bacterial blight resistance, namely the *Xa21*, which belongs to the *R* gene group and is originated from the wild rice species *Oryza longistaminata* (Ni et al. 2015). Another example is the *R* gene *Pi54* that was identified in both wild and cultivated rice and shown to play a role in blast disease resistance (Zhang et al. 2018). Sources of common wild rice, however, are becoming rare due to human activities. China has protected several conservation areas to maintain the production of wild rice and preserve its genetic diversity for rice breeding efforts, as well as to provide research materials to investigate the responses of wild and cultivated varieties of rice to various abiotic and biotic stresses (Luo et al. 2017; Tian et al. 2017).

Mycorrhizae are well known for their symbiotic associations with host plants (Grove et al. 2017; Verzeaux et al. 2017; Jemo et al. 2018). More than 80% of plant species can be colonized by arbuscular mycorrhizal (AM) fungi (AMFs), which develop an endosymbiosis with their host (Feddermann et al. 2010). AMFs, among other attributes, improve the ability of host plants to capture nutrients from the soil (Grove et al. 2017; Verzeaux et al. 2017), and the fundamental aspect of the AMF symbiosis with host plants is the bidirectional exchange of nutrients (Field and Pressel 2018; Karandashov and Bucher 2005). The improvement in nutrient uptake (e.g. phosphorus) (Berdeni et al. 2018; Selvakumar et al. 2018) from soil by host plants has been reported to result from the generation of long hyphae into the soil around plant roots and the ability of AMFs to increase resistance of host plants to environmental stressors (Jones et al. 2004; Berdeni et al. 2018; Selvakumar et al. 2018; Tian et al. 2019). In turn, AMFs can obtain carbon (C) nutrients (photosynthates) from the host plants to grow and survive (Tian et al. 2010; Zhang et al. 2016). Rice domestication has been reported to have substantially changed the benefits derived from AMFs (Martín-Robles et al. 2018), suggesting that the AM mechanisms and reactions occurring in wild rice may be different than what occurs in cultivated rice. Increasing number of research has demonstrated that AMFs can improve resistance of rice plants to various pathogenic fungi, including *M. oryzae* (Baby 2001; Campos-Soriano et al. 2012). However, no detailed comparative studies have been conducted on the response of wild AM rice vs. cultivated AM rice during *M. oryzae* infection.

Although the existence of disease resistance in common wild rice has been well established (Liu et al. 2017; Stein et al. 2018), only a few studies have been conducted which demonstrate that wild rice selectively chooses and closely connects with a beneficial microbiome (Martín-Robles et al. 2018; Perez-Jaramillo et al. 2018; Shi et al. 2018a). The resistance of wild rice to *M. oryzae* may be partially attributable to the presence of its symbiotic AMFs (Martín-Robles et al. 2018), while the susceptibility of cultivated rice to pathogenic fungi may have arisen due to the change in the original AMF community occurred during the domestication of wild rice (Thomas et al. 2017). For instance, a recent study reported that several AMF taxa within the Glomeromycota were more enriched in wild rice in comparison with cultivated rice in the presence of *M. oryzae*; and thus, the enrichment of Glomeromycota was suggested to be correlated with the higher resistance of wild rice to *M. oryzae* (Shi et al. 2018a)*.* These findings provided an insight into the relationship between wild and cultivated rice with regard to their associated AMFs in response to *M. oryzae*. The identified AMFs have been shown to stimulate metabolic pathways involved in disease resistance in wild rice (Shi et al. 2018a), suggesting that they are involved in promoting plant resistance to *M. oryzae*. It is then interesting to determine the mechanisms by which the identified AMFs, including the *Rhizoglomus* spp*.*, benefit rice under the background of domestication when plants are infected by *M. oryzae*.

In the present study, experiments were conducted to assess the response of *R. intraradices*-colonizing wild and cultivated rice to *M. oryzae* to provide information on how this inoculated AMF species could contribute to the resistance of wild rice, and examine whether this *R. intraradices* could enhance the resistance of cultivated rice to *M. oryzae*. We hypothesized that 1) wild AM rice has retained some symbiotic benefits that have been lost during the domestication of cultivated AM rice which enhance resistance to *M. oryzae*, and unraveled that 2) the molecular mechanisms that the AM fungus *R. intraradices* used to enhance resistance to *M. oryzae* differed between wild and cultivated rice.

## Methods

### Plant materials and experimental design

Seedlings of cultivated rice (*Oryza sativa* L. ssp. Japonica) and Dongxiang wild rice (a Chinese common wild rice; *Oryza rufipogon* Griff.) (Li et al. 2010) were used in this study. *M. oryzae* was cultured on an oatmeal agar medium, and hyphae were “brushed” to induce the formation of conidia as described by Chumley and Valent (1990) and Koga et al. (2004).

The *R. intraradices* strain (BGC BJ09) used in this study was provided by the Institute of Plant Nutrition and Resources, Beijing Academy of Agriculture and Forestry in China (Liu et al. 2014). Spores of the AMF *R. intraradices* were obtained from the soil of *Medicago sativa* plants grown in pots that were previously inoculated with *R. intraradices* for 5 months. Briefly, spores were collected from the mycorrhizosphere of *M. sativa* using a wet-sieving method that involved placing the mycorrhizosphere in a 45-μm mesh sieve and sufficiently rinsing them with tap water at room temperature. The collected spores were then diluted to a concentration of ~ 10 spores/mL. The spores were surface-sterilized in 0.5% sodium hypochlorite for 3 min and rinsed twice in sterile deionized water prior to their use in the inoculation of the rice seedlings (Green et al. 1976).

Rice seeds were surface-sterilized with 1% sodium hypochlorite for 5 min and incubated in a dark chamber at 25 °C for 3 d. Six germinated seeds were then transplanted into each pot [18 (width) × 18 (height) cm] containing soil. The soil was collected from the experiment station of Northeast Institute of Geography and Agroecology, and was autoclaved before use. The soil characteristics were analyzed and reported previously in Tian et al. (2018) as follows: soil organic matter 61.2 g/kg, total nitrogen 651.92 mg/kg, available-nitrogen 109.20 mg/kg, available-phosphorus 7.48 mg/kg, and available-potassium 88.66 mg/kg, and pH 6.31. The pots were maintained in a growth chamber that was preset to 16-h-light (1000 μmol/m^2^/s)/8-h-dark photoperiod, 23–28 °C and 65% relative humidity. Seven days later, three plants with similar size were kept in each pot. For treatment with *R. intraradices*, the 10-day-old wild and cultivated rice plants were inoculated with *R. intraradices* by pouring 200 mL of its spores (~ 10 spores/mL) to each pot (W + R and C + R). Non-treated wild rice (W) and cultivated rice (C) plants were used as controls. The pots were incubated in a chamber under the light and temperature cycle described above for 45 d to guarantee the AM colonization.

**Fig. 1:**
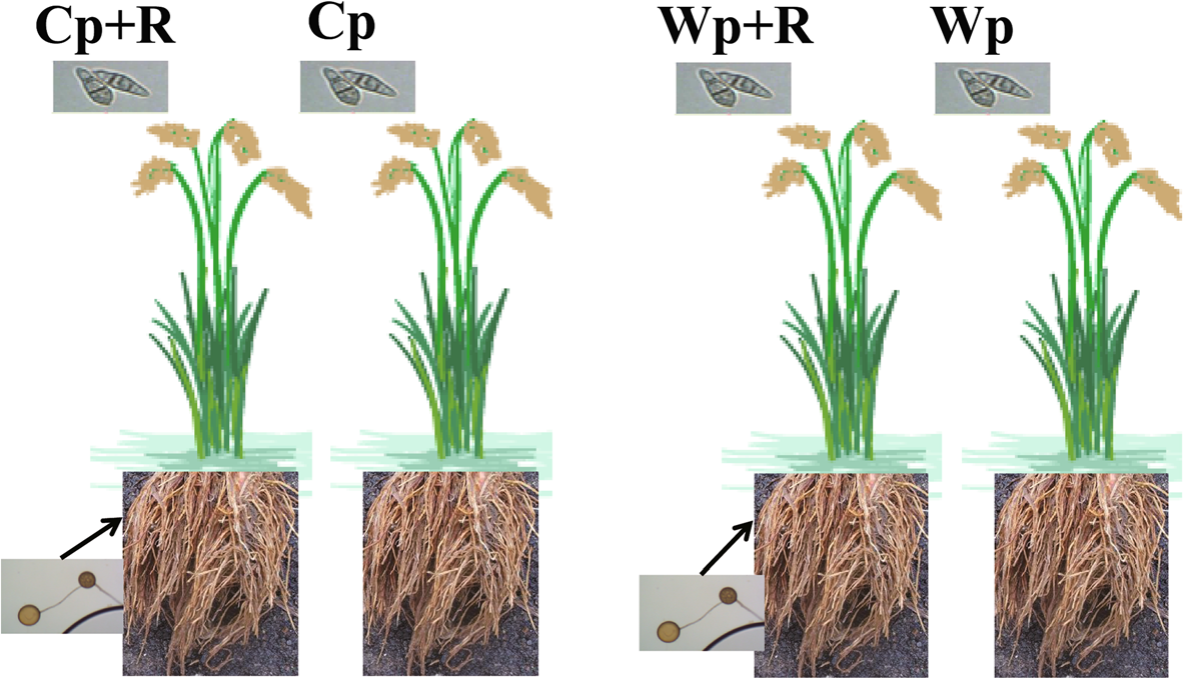
The four experimental treatment groups designed to comparatively study the responses of wild and cultivated rice plants to *Magnaporthe oryzae* infection with and without inoculation with the arbuscular mycorrhizal fungus *Rhizoglomus intraradices*. Cp + R, *R. intraradices*-inoculated cultivated rice infected with *M. oryzae*; Cp, *R. intraradices*-uninoculated cultivated rice infected with *M. oryzae*; Wp + R, *R. intraradices*-inoculated wild rice infected with *M. oryzae*; Wp, *R. intraradices*-uninoculated wild rice infected with *M. oryzae*

The primary focus of the experiment was to examine the differences between wild and cultivated rice inoculated with the AMF *R. intraradices* in response to the infection with *M. oryzae* pathogen (p). Thus, after 45 d of growth, all four groups (W + R, C + R, W and C) were infected with the pathogenic fungus *M. oryzae*. Spores of *M. oryzae* with the concentration of 10^6^/mL were evenly sprayed on the leaves of individual plants, and the plants were subsequently incubated in a chamber in the dark at 80% humidity and 25 °C for 2 d. Consequently, we obtained four treatment groups for this study: *R. intraradices*-inoculated cultivated rice infected with *M. oryzae* (Cp + R), *R. intraradices*-uninoculated cultivated rice infected with *M. oryzae* (Cp), *R. intraradices*-inoculated wild rice infected with *M. oryzae* (Wp + R), *R. intraradices*-uninoculated wild rice infected with *M. oryzae* (Wp) (see Fig. 1 for the scheme of experimental design). The plants of all four treatment groups (Wp + R, Cp + R, Wp and Cp) were then maintained in the chambers under the same growth conditions [8 h dark at 23 °C and 16 h light (1000 μmol/m^2^/s) at 28 °C] for a total of 7 d prior to sampling.

### Sampling and phenotyping

Seven days after plants were infected with *M. oryzae* spores, leaves were sampled and used to analyze the leaf phenotype. The ability of the plants in resisting *M. oryzae* was analyzed using the typical injured leaves by using the Image J software (v.1.52i) based on lesion areas (Campos-Soriano et al. 2012). Root samples were also extracted from the soil and divided into parts. One part was cleaned with tap water and immediately placed in liquid nitrogen and stored at − 80 °C for RNA extraction. Another part of roots along with their rhizosphere was used to evaluate the presence of *R. intraradices*. The plant growth phenotype was recorded prior to any leaf or root sampling. Lastly, rhizosphere soils were sampled and kept at − 80 °C until DNA extraction.

### Root colonization rate of *R*. *intraradices*, and determination of *R*. *intraradices* levels in the rhizosphere soil by real-time quantitative PCR (qPCR)

Root colonization rate of *R. intraradices* was measured by the trypan blue method as described by Phillips and Hayman (1970) and Tian et al. (2019). The method described by Trouvelot et al. (1986) was used for the calculation of the arbuscule abundance in mycorrhizal parts of root fragments. For determination of the abundance of *R. intraradices* propagating in the rhizosphere soil during rice growth, total DNA was extracted from rhizosphere soil as previously described by Tian et al. (2018), and was then used to analyze the 18S ribosomal DNA (rDNA) gene expression by qPCR (Dai et al. 2015). Prior to the qPCR, the quality and concentrations of purified DNA samples were determined using a NanoDrop 2000 (NanoDrop Technologies, Inc., Wilmington, USA). The *R. intraradices* abundance was expressed as the copy number of the 18 s rDNA region of each fungus per one gram soil, which was amplified using the forward primer AMV4.5NF (5′-AAGCTCGTAGTTGAATTTCG-3′) and reverse primer AMDGR (5′-CCCAACTATCCCTATTAATCAT-3′) (Sato et al. 2005).

### RNA extraction, cDNA library construction and RNA-sequencing

Roots from three biological replicates from each treatment group were collected and subjected to RNA extraction as described previously (Tian et al. 2018). The quality and concentration of extracted RNA samples were assessed spectrophotometrically using a NanoDrop 2000 (NanoDrop Technologies, Inc., Wilmington, USA). cDNA libraries were subsequently constructed as described by Chen et al. (2017) (Chen et al. 2017). For RNA-sequencing (RNA-seq), paired-end sequencing (2 × 100 bp) was conducted using an Illumina HiSeq X Ten platform (Illumina, San Diego CA, USA) at the Novogene Co., Ltd. (Beijing, China). FastQC (http://www.bioinformatics.babraham.ac.uk/projects/fastqc/) and Cutadapt (http://cutadapt.readthedocs.io/en/stable/) were used to determine the sequence quality. Filtered reads (~ 28 million) were mapped onto the rice reference genome using bowtie with default settings (http://bowtie-bio.sourceforge.net/index.shtml). A comparative analysis of gene expression was used to identify differentially expressed genes (DEGs). Cufflinks (http://sihua.us/Cufflinks.htm) was used to conduct a *t*-test (*P* < 0.05) and to identify DEGs in the *R. intraradices*-inoculated compared with the *R. intraradices*-non-inoculated roots of cultivated (‘Cp+R vs. Cp’) and wild rice (‘Wp+R vs. Wp’) with and without *M. oryzae*. A false discovery rate (FDR) of 5% (*q*-value < 0.05) was used to identify DEGs with at least a |log_2_ (fold-changes)| > 0. Gene ontology (GO) annotations were performed using Blast2GO v2.5 based on the nonredundant (Nr) protein sequences (NCBI) and Pfam (NCBI, nonredundant nucleotide sequences). A total of 20,693 annotated genes with known functions were included in the GO annotation and enrichment analyses. The KEGG Automatic Annotation Server (KAAS; http://www.genome.jp/kegg/kaas/) was used for KEGG annotations. MapMan analysis was conducted using MapMan 3.6.0 (http://mapman.gabipd.org/web/guest) software.

### Validation of RNA-seq data by reverse transcription-quantitative real-time PCR (RT-qPCR)

To validate the quality of the RNA-seq analysis, RT-qPCR analysis was conducted on eight selected genes, namely *Os12g0168700*, *Os06g0726200*, *Os04g0229100*, *Os02g0627100*, *Os01g0854800*, *Os01g0892500*, *Os02g0175000* and *Os02g0678200*, with the *β-tubulin* gene of *O. sativa* being used as a reference gene (Liu et al. 2009), as described previously (Tian et al. 2018). The gene-specific primers utilized in the RT-qPCR analysis are listed in Additional file 1: Table S1. Among the eight selected genes, *Os12g0168700*, *Os06g0726200*, *Os04g0229100*, *Os02g0627100* and *Os01g0854800* were DEGs in the ‘Wp+R vs. Wp’ comparison, while *Os01g0892500*, *Os02g0175000* and *Os02g0678200* were DEGs in the ‘Cp+R vs. Cp’ comparison.

### Statistical analysis

SPSS 19.0 software was used for one-way analysis of variance to determine the statistical significance of the *R. intraradices* colonization rate and the abundance of *R. intraradices* propagating in the rhizosphere soil during rice growth, as well as for the statistical analysis of gene expression data (Student’s *t*-test).

## Results

### Colonization rate of inoculated *R. intraradices* and abundance of the *R. intraradices* propagating in the rhizosphere soil during rice growth

**Fig. 2:**
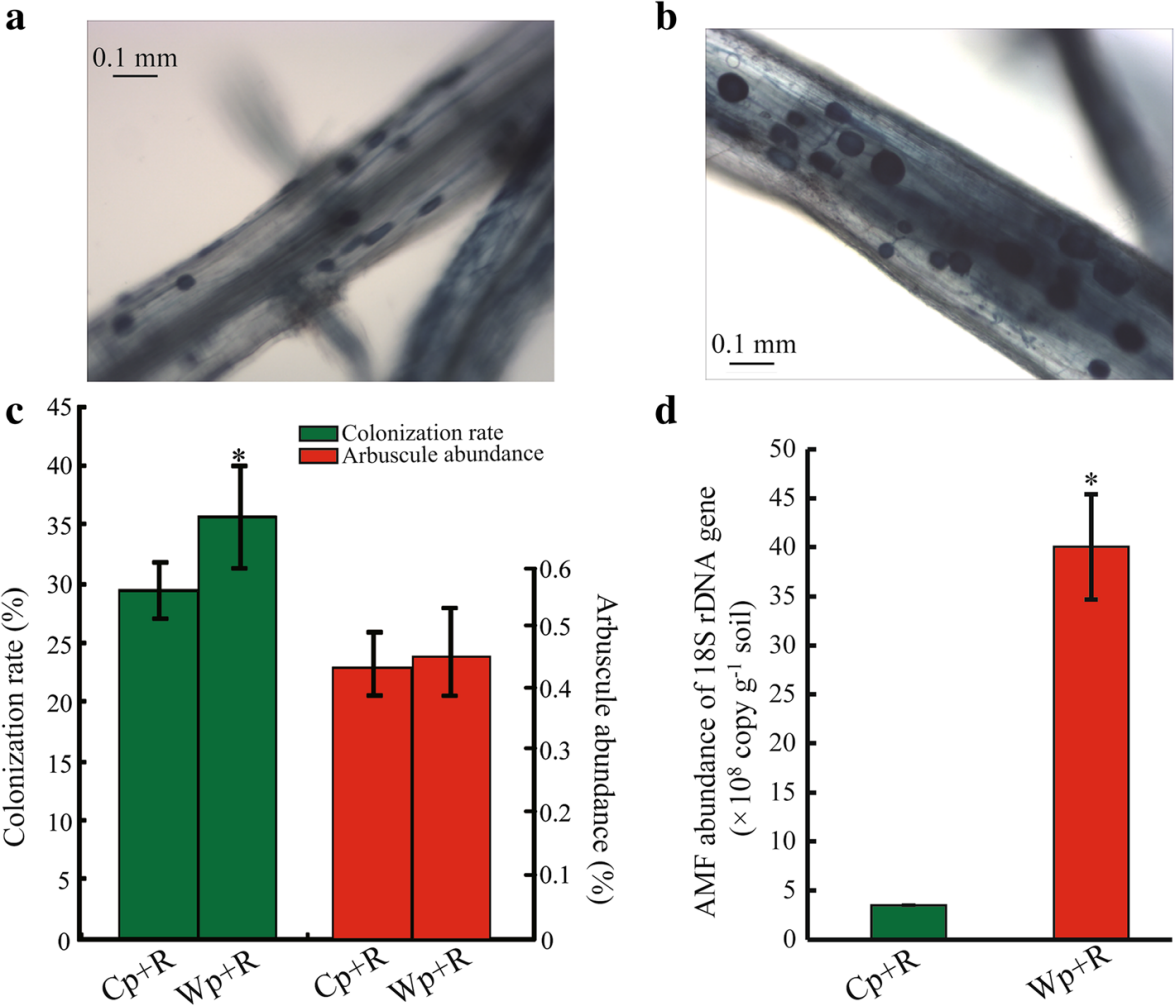
**a-b** Microscopic observation of root colonization of (**a**) wild and (**b**) cultivated rice inoculated with the arbuscular mycorrhizal fungus *Rhizoglomus intraradices*. **c-d** Colonization rate and arbuscule abundance of *R. intraradices* in roots of inoculated rice plants (**c**) and abundance of *R. intraradices* (**d**) in the rhizosphere soil of rice plants following soil inoculation with *R. intraradices* and plant infection with *Magnaporthe oryzae*. Data shown are the *R. intraradices* abundance in Cp + R and Wp + R. The error bars represent standard deviations of the means of three repeats. The asterisks above the bars indicate significant differences among the samples at *P* < 0.05 based on Student’s *t*-test. Cp + R, *R. intraradices*-inoculated cultivated rice infected with *M. oryzae*; Wp + R, *R. intraradices*-inoculated wild rice infected with *M. oryzae*

The colonization of cultivated and wild rice by *R. intraradices* was quantified using the trypan blue method (Phillips and Hayman 1970; Tian et al. 2019). Results indicated that *R. intraradices* was able to colonize both wild and cultivated rice (Fig. 2a and b). Investigation of colonization rate of roots with inoculated *R. intraradices* showed that Wp + R had a higher colonization rate than Cp + R after inoculation with *R. intraradices* and infection with *M. oryzae* (Fig. 2c). However, although Wp + R showed a slightly higher arbuscule abundance of *R. intraradices* than Cp + R, this difference is statistically insignificant, perhaps due to the very low levels of arbuscule abundance detected (Fig. 2c). The levels of *R. intraradices* propagating in the rhizosphere soil were also quantified using qPCR. Results indicated that the abundance of soil *R. intraradices* was significantly higher in the wild rice rhizosphere (roots plus surrounding soil) than in the cultivated rice rhizosphere (Fig. 2d), indicating that the wild rice rhizosphere could harbor more *R. intraradices* than the cultivated rice rhizosphere under our experimental conditions.

### The effect of *R*. *intraradices* colonization on the response of rice plants to pathogen infection

**Fig. 3:**
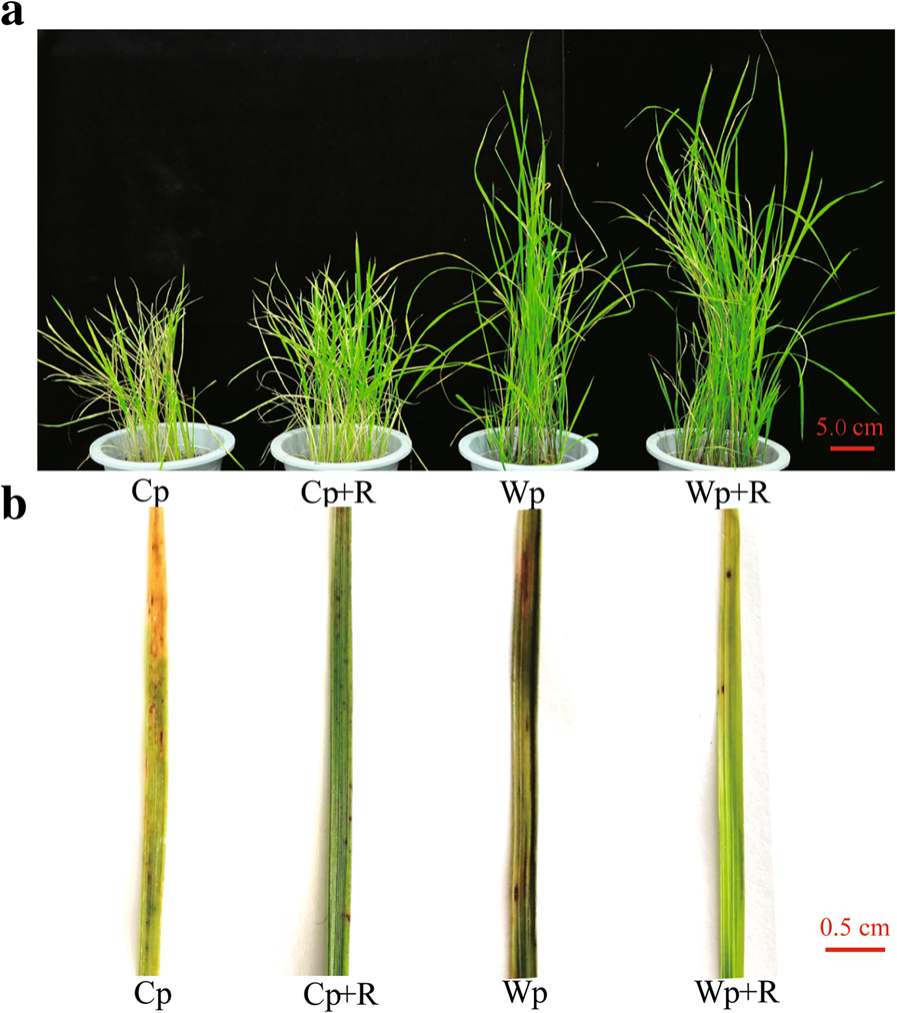
Phenotypes of wild and cultivated rice plants in response to *Magnaporthe oryzae* infection with and without inoculation with the arbuscular mycorrhizal fungus *Rhizoglomus intraradices*. **a** plant growth and (**b**) infected leaves. Cp + R, *R. intraradices*-inoculated cultivated rice infected with *M. oryzae*; Cp, *R. intraradices*-uninoculated cultivated rice infected with *M. oryzae*; Wp + R, *R. intraradices*-inoculated wild rice infected with *M. oryzae*; Wp, *R. intraradices*-uninoculated wild rice infected with *M. oryzae*. Red lines are the scale bars, which represent 5.0 cm in (**a**) and 0.5 cm in **(b**)

Examination of the growth and disease symptoms of the wild and cultivated rice plants revealed that the Wp were more resistant to *M. oryzae* than the Cp plants (with 19.1% and 25.73% lesion areas in Wp and Cp, respectively) (Fig. 3; Additional file 2: Figure S1), and Wp + R were more resistant to *M. oryzae* than the Cp + R plants (with 4.77% and 7.87% lesion areas in Wp + R and Cp + R, respectively) (Fig. 3; Additional file 2: Figure S1). Notably, both the wild and cultivated rice plants inoculated with *R*. *intraradices* (Wp + R and Cp + R) exhibited a greater resistance to infection by the rice blast fungus than their corresponding Wp or Cp plants that were non-inoculated with *R*. *intraradices* (Fig. 3; Additional file 2: Figure S1). In all four treatment groups Wp + R, Wp, Cp + R and Cp, the leaf surfaces of all rice plants exhibited obvious lesion areas; however, the total lesion areas were significantly smaller in Wp + R, followed by that of Cp + R, Wp and Cp plants, suggesting the positive effect of *R. intraradices* in providing resistance against *M. oryzae* (Fig. 3b; Additional file 2: Figure S1).

### Comparative analysis of the responses of cultivated and wild rice roots using the transcriptomic approach

**Fig. 4:**
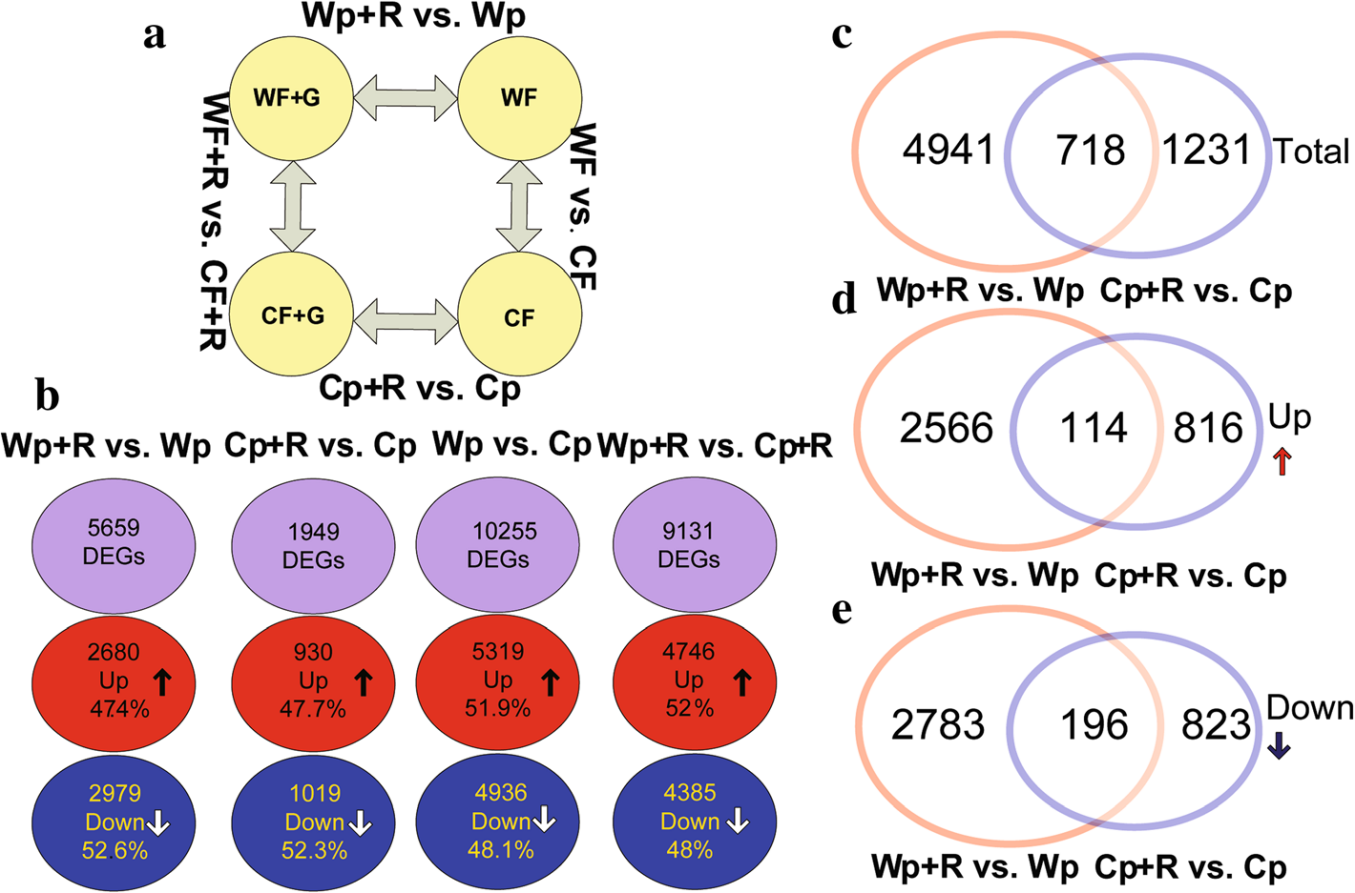
Numbers of differentially expressed genes (DEGs). **a** Diagram illustrating the experimental design and comparisons. **b** Diagram illustrating the number of total, up-regulated, and down-regulated genes in the four comparisons. **c**, **d**, and **e**. A Venn analysis of the number of total, up-regulated, and down-regulated DEGs identified in the ‘Wp+R vs. Wp’ and ‘Cp+R vs. Cp’ comparisons. Cp + R, *Rhizoglomus intraradices*-inoculated cultivated rice infected with *Magnaporthe oryzae*; Cp, *R. intraradices*-uninoculated cultivated rice infected with *M. oryzae*; Wp + R, *R. intraradices*-inoculated wild rice infected with *M. oryzae*; Wp, *R. intraradices*-uninoculated wild rice infected with *M. oryzae*

**Fig. 5:**
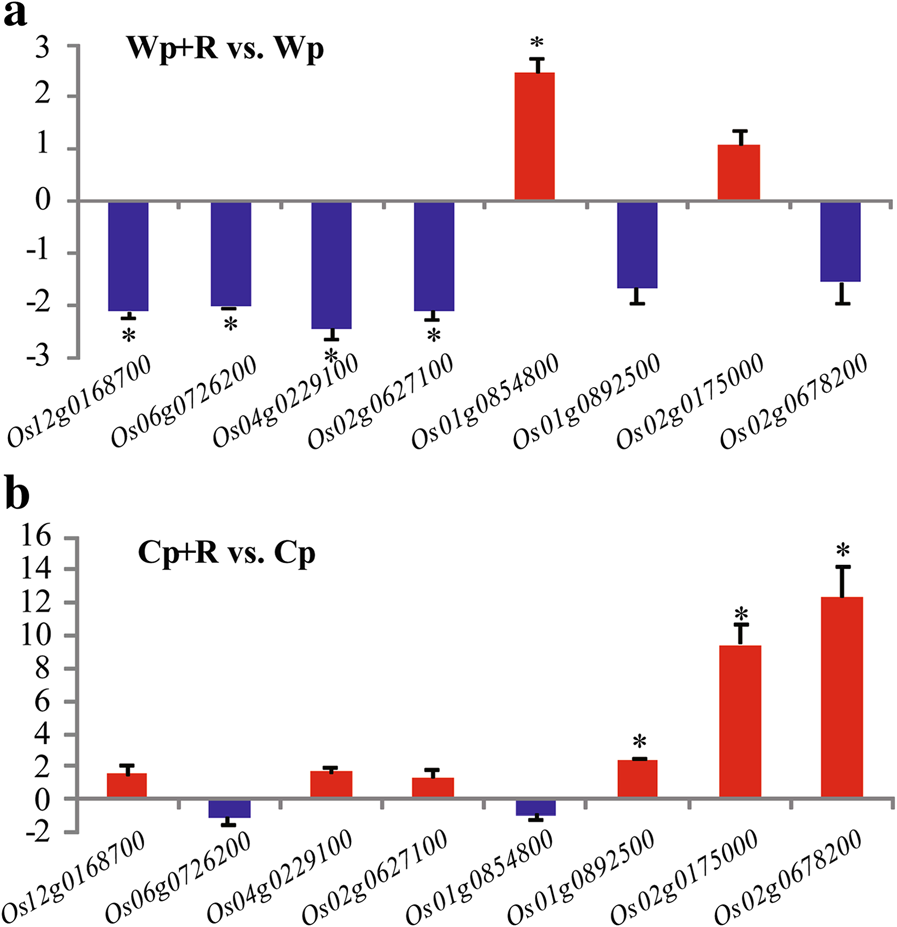
Validation of the RNA-sequencing data using reverse transcription-quantitative real-time PCR (RT-qPCR). Eight genes were selected for RT-qPCR analysis from the RNA-sequencing data. **a** Relative expression of the selected genes in the ‘Wp+R vs. Wp’ comparison. **b** Relative expression in the ‘Cp+R vs. Cp’ comparison. Cp + R, *Rhizoglomus intraradices*-inoculated cultivated rice infected with *M. oryzae*; Cp, *R. intraradices*-uninoculated cultivated rice infected with *M. oryzae*; Wp + R, *R. intraradices*-inoculated wild rice infected with *M. oryzae*; Wp, *R. intraradices*-uninoculated wild rice infected with *M. oryzae*. The error bars represent standard deviations of the means. The asterisks above the bars indicate significant change in gene expression at *P* < 0.05 based on Student’s *t*-test

The root transcriptomes of wild and cultivated rice varieties inoculated and non-inoculated with *R. intraradices* were compared to elucidate their molecular response to infection by the rice blast fungus. A total of ~ 624 million raw reads were obtained from the 12 samples, with the number of reads ranging from 30.54 to 64.75 million raw reads in each sample (Table 1). After filtering out low quality reads, a total of ~ 556 million clean reads were obtained with an average of 84.18% that could be mapped to the rice reference genome Oryza_sativa.IRGSP-1.0.21 (http://asia.ensembl.org). The percentage of clean reads (144.4–146 bp in average length) from each sample that could be mapped ranged from 74.76 to 89.35% (Table 1). A total of 5659 (2680 up- and 2979 down-regulated) genes were identified as being differentially expressed genes (DEGs) in the ‘Wp+R vs. Wp’ comparison, while a total of 1949 DEGs were identified in the ‘Cp+R vs. Cp’ comparison (Fig. 4). Subsequently, 8 genes were selected and subjected to RT-qPCR analysis to confirm the results of the RNA-seq analysis. Results demonstrated that the RT-qPCR data were in general consensus with the RNA-seq data (Fig. 5; Additional file 3: Table S2).

**Table 1:**
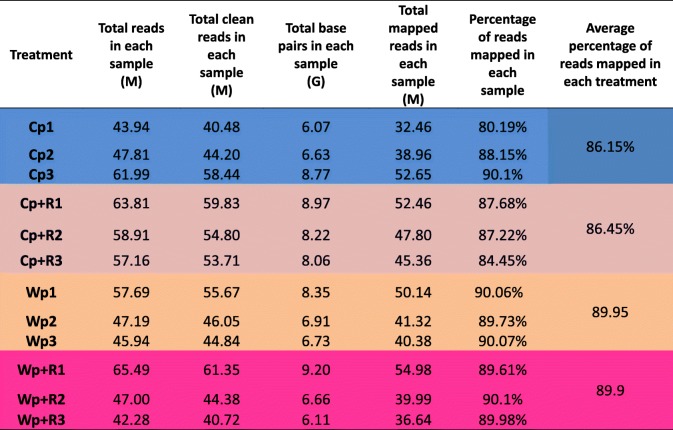
Summary of Illumina RNA-sequencing reads mapped to the reference genome. Cp + R, *Rhizoglomus intraradices*-inoculated cultivated rice infected with *Magnaporthe oryzae*; Cp, *R. intraradices*-uninoculated cultivated rice infected with *M. oryzae*; Wp + R, *R. intraradices* -inoculated wild rice infected with *M. oryzae*; Wp, *R. intraradices*-uninoculated wild rice infected with *M. oryzae*. M, million; G, gbase

### Gene ontology (GO) annotation and enrichment analysis of the identified DEGs

The DEGs of the ‘Wp+R vs. Wp’ and ‘Cp+R vs. Cp’ comparisons were subjected to GO analysis to assign functional terms to the identified DEGs. The top 20 enriched GO categories were then used to determine differences between the ‘Wp + R vs. Wp’ and ‘Cp+F vs. Cp’ comparisons. There were 12, 5, and 3 GO categories in the ‘Wp + R vs. Wp’ comparison labeled as ‘biological_process’, ‘cellular_component’, and ‘molecular_function’, respectively (Table 2). The GO analysis of the DEGs derived from ‘Wp + R vs. Wp’ comparison indicated that within ‘biological_process’, the terms ‘response to stimulus’, ‘biological regulation’, ‘regulation of biological process’, ‘regulation of cellular process’, ‘developmental process’, ‘single-organism developmental process’, ‘cellular response to stimulus’, ‘cell communication’, ‘response to organic substance’, ‘single organism signaling’, ‘signaling’ and ‘signal transduction’ were enriched (Table 2). As for the ‘cellular_component’ category, ‘cell’, ‘cell part’, ‘cellular_component’, ‘nucleus’ and ‘cell periphery’ were enriched in this category, whereas ‘binding’, ‘nucleic acid binding’, and ‘protein binding’ were enriched in the ‘molecular_function category’ (Table 2).

**Table 2:**
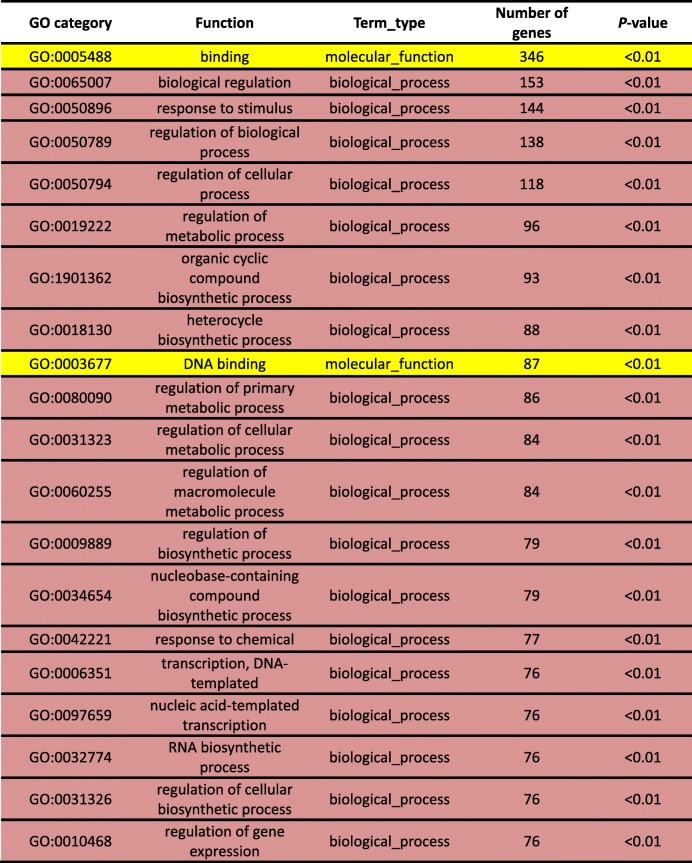
Gene ontology (GO) classification of differentially expressed genes (DEGs) derived from the ‘Wp+R vs. Wp’ comparison. Wp + R, *Rhizoglomus intraradices*-inoculated wild rice infected with *Magnaporthe oryzae*; Wp, *R. intraradices*-uninoculated wild rice infected with *M. oryzae*

Comparatively, the GO analysis of the DEGs obtained from ‘Cp+F vs. Cp’ comparison resulted in 18 enriched GO terms within the ‘biological_process’ category and 2 enriched GO terms within the ‘molecular_function’ category (Table 3). Specifically, the enriched terms within the ‘biological_process’ were ‘biological regulation’, ‘response to stimulus’, ‘regulation of biological process’, ‘regulation of cellular process’, ‘regulation of metabolic process’, ‘organic cyclic compound biosynthetic process’, ‘heterocycle biosynthetic process’, ‘regulation of primary metabolic process’, ‘regulation of cellular metabolic process’, ‘regulation of macromolecule metabolic process’, ‘regulation of biosynthetic process’, ‘nucleobase-containing compound biosynthetic process’, ‘response to chemical’, ‘transcription, DNA-templated’, ‘nucleic acid-templated transcription’, ‘RNA biosynthetic process’, ‘regulation of cellular biosynthetic process’ and ‘regulation of gene expression’ (Table 3), while the 2 enriched GO terms within the ‘molecular_function’ category were ‘binding’ and ‘DNA binding’ (Table 3).

**Table 3:**
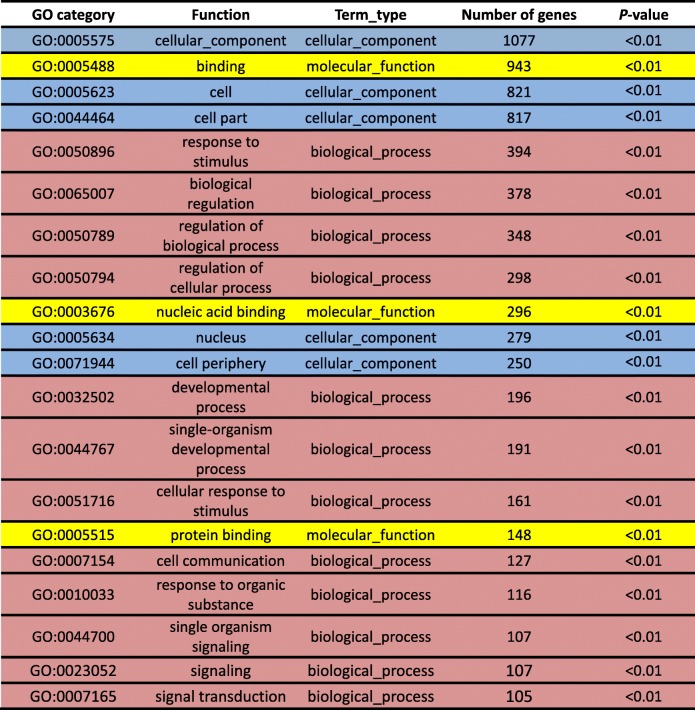
Gene ontology (GO) classification of the differentially expressed genes (DEGs) derived from the ‘Cp+R vs. Cp’ comparison. Cp + R, *Rhizoglomus intraradices*-inoculated cultivated rice infected with *Magnaporthe oryzae*; Cp, *R. intraradices*-uninoculated cultivated rice infected with *M. oryzae*

### KEGG analysis of the identified DEGs

A KEGG analysis of the ‘Wp+R vs. Wp’ comparison revealed that the pathways of ‘spliceosome valine’, ‘leucine and isoleucine degradation’, ‘fatty acid degradation’, ‘plant hormone signal transduction’, ‘phenylalanine metabolism’ and ‘α-linolenic acid metabolism’ were significantly enriched (Table 4; Additional file 4: Table S3a). It is noteworthy that α-linolenic acid metabolism is related to jasmonic acid (JA) synthesis, and JA, as one of the major biotic stress-related phytohormones, may help to induce plant induced systemic resistance (ISR) (Iwanami et al. 2017). Further analysis showed that 9 α-linolenic acid metabolism-related genes (e.g., *Os11g0210300* and *Os06g0346300*) were up-regulated in the ‘Wp + R vs. Wp’ comparison, while no α-linolenic acid metabolism-related genes were induced in the ‘Cp+R vs. Cp’ comparison (Table 5). Comparatively, the pathways of ‘plant hormone signal transduction’, ‘brassinosteroid biosynthesis’, ‘tropane, piperidine and pyridine alkaloid biosynthesis’, ‘tyrosine metabolism’, ‘glutathione metabolism’, ‘glycine, serine and threonine metabolism’, ‘glycerolipid metabolism’ and ‘thiamine metabolism’ were significantly enriched in the ‘Cp + R vs. Cp’ comparison (Table 6; Additional file 4: Table S3b). Brassinosteroids are a group of steroid hormones with important regulatory function in plant responses to both biotic and abiotic stresses (Bari and Jones 2009; Choudhary et al. 2012), and brassinosteroids-related genes (e.g., *Os03g0227700*, *Os05g0200400* and *Os04g0469800*) were up-regulated only in the ‘Cp + R vs. Cp’ comparison (Table 5; Table 6). Only the pathway ‘plant hormone signal transduction’ was shared between the ‘Wp + R vs. Wp’ (with 32 genes up-regulated) and ‘Cp + R vs. Cp’ (with 18 genes up-regulated) comparisons (Additional file 4: Table S3a and b). A more detailed analysis of the functions of the plant hormone-related genes revealed that auxin-related genes were up-regulated among the hormone-related genes common to both the ‘Wp + R vs. Wp’ and ‘Cp + R vs. Cp’ comparisons.

**Table 4:** KEGG analysis of the response of *Rhizoglomus intraradices*-inoculated wild rice plants to infection by *Magnaporthe oryzae* in the ‘Wp+R vs. Wp’ comparison. Wp + R, *R. intraradices*-inoculated wild rice infected with *M. oryzae*; Wp, *R. intraradices*-uninoculated wild rice infected with *M. oryzae*

Term	Number of genes	*P*-value
Spliceosome	32	< 0.01
Valine, leucine and isoleucine degradation	10	0.01
Fatty acid degradation	10	0.02
Plant hormone signal transduction	32	0.02
Phenylalanine metabolism	24	0.02
α-linolenic acid metabolism	9	0.04

**Table 5:** List of several important differentially expressed genes derived from W + F versus W (Wp + R vs. Wp) comparison and Cp + R versus C (Cp + R vs. C) comparison. Cp + R, *Rhizoglomus intraradices*-inoculated cultivated rice infected with *M. oryzae*; Cp, *R. intraradices*-uninoculated cultivated rice infected with *M. oryzae*; Wp + G, *R. intraradices*-inoculated wild rice infected with *M. oryzae*; Wp, *R. intraradices*-uninoculated wild rice infected with *M. oryzae*

Gene ID	Description	RNA-sequencing data			
		Wp + G vs. Wp log_2_(fold-change)	q-value	Cp + G vs. Cp log_2_(fold-change)	q-value
*Os11g0210300*	α-linolenic acid metabolism-related genes	1.62	< 0.01	unchanged	
*Os06g0346300*	α-linolenic acid metabolism-related genes	2.99	< 0.01	unchanged	
*Os03g0227700*	brassinosteroids-related genes	−1.30	< 0.01	1.41	< 0.01
*Os05g0200400*	brassinosteroids-related genes	−0.61	< 0.01	0.79	< 0.01
*Os04g0469800*	brassinosteroids-related genes	unchanged		0.74	0.03
*Os05g0418100*	Mildew Resistance Locus O (MLO)-like proteins	1.10	< 0.01	unchanged	
*Os11g0181400*	Mildew Resistance Locus O (MLO)-like proteins	1.11	< 0.01	unchanged	
*Os08g0244500*	encoding 1,3-ß-glucosidases	4.36	< 0.01	unchanged	
*OS01g0713200*	encoding 1,3-ß-glucosidases	2.75	< 0.01	unchanged	
*Os07g0600700*	endo-1,3-ß-glucosidases	unchanged		1.68	< 0.01
*Os09g0422500*	cell wall-related genes	5.23	< 0.01	unchanged	
*Os01g0750300*	cell wall-related genes	2.31	< 0.01	unchanged	
*Os01g0746700*	cell wall-related genes	1.76	< 0.01	unchanged	
*Os08g0345500*	cellulose synthases	−2.16	0.04	1.46	< 0.01
*Os08g0160500*	cellulose synthases	unchanged		1.20	< 0.01
*Os09g0428000*	cellulose synthases	unchanged		0.84	0.01
*Os02g0218700*	allene oxide synthase and lipoxygenase	0.73	0.01	−3.05	< 0.01
*Os03g0738600*	allene oxide synthase and lipoxygenase	1.35	0.02	unchanged	
*Os11g0256900*	carboxyl methyltransferase	0.68	< 0.01	unchanged	
*Os04g0665200*	carboxyl methyltransferase	2.27	< 0.01	unchanged	
*Os11g0260100*	carboxyl methyltransferase	−1.04	< 0.01	1.34	< 0.01
*Os01g0701700*	carboxyl methyltransferase	−0.73	0.03	1.65	< 0.01
*Os01g0375500*	encoding a shikimate 5-dehydrogenase	2.27	0.04	unchanged	
*Os03g0118800*	mevalonate pathway (MVA)-related gene	−1.73	< 0.01	unchanged	
*Os09g0492700*	mevalonate pathway (MVA)-related gene	0.67	< 0.01	unchanged	
*OS07G0101000*	simple phenols-related gene	1.72	0.01	unchanged	
*OS05G0458300*	simple phenols-related gene	2.89	< 0.01	unchanged	
*OS02G0749700*	phenols-related gene	unchanged		−5.05	< 0.01
*OS12G0258700*	phenols-related gene	unchanged		−2.23	0.04
*Os02g0570400*	terpenoid pathway-related gene	2.82	0.02	−2.77	< 0.01
*Os04g0344100*	terpenoid pathway-related gene	1.53	< 0.01	unchanged	
*Os02g0568700*	terpenoid pathway-related gene	unchanged		−5.34	< 0.01
*Os02g0571100*	terpenoid pathway-related gene	4.96	< 0.01	−4.11	< 0.01

**Table 6:** KEGG analysis of the response of *Rhizoglomus intraradices*-inoculated cultivated rice plants to infection by *Magnaporthe oryzae* in the Cp + R vs. Cp comparison. Cp + R, *R. intraradices*-inoculated cultivated rice infected with *M. oryzae*; Cp, *R. intraradices*-uninoculated cultivated rice infected with *M. oryzae*

Term	Number of genes	*P*-value
Plant hormone signal transduction	18	< 0.01
Brassinosteroid biosynthesis	3	0.01
Tropane, piperidine and pyridine alkaloid biosynthesis	4	0.01
Tyrosine metabolism	5	0.01
Glutathione metabolism	7	0.02
Glycine, serine and threonine metabolism	6	0.02
Glycerolipid metabolism	5	0.03
Thiamine metabolism	2	0.04

### Identification of biotic stress-related genes among the identified DEGs

**Fig. 6:**
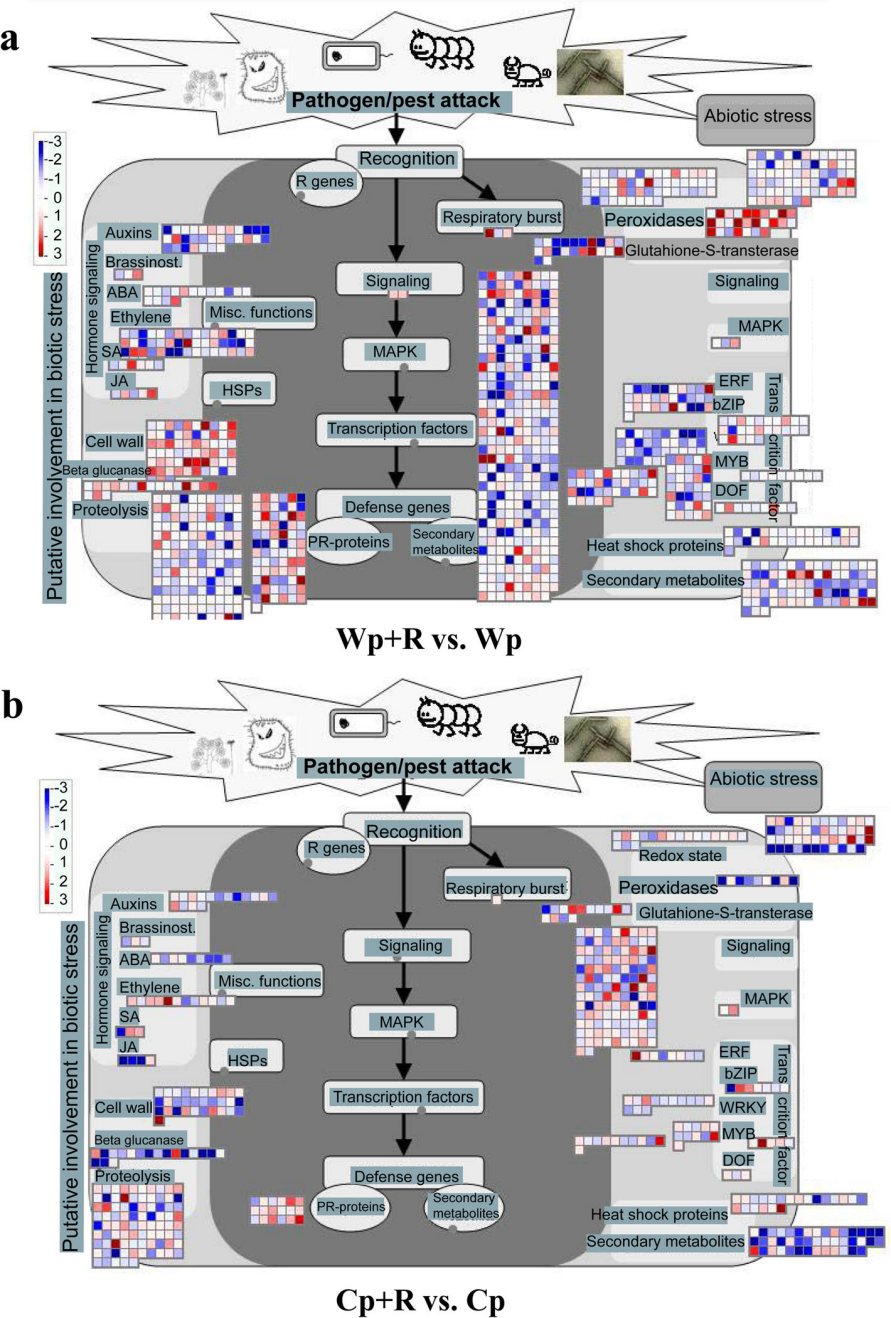
Analysis of the biotic stress response of *Rhizoglomus intraradices*-inoculated plants to infection by *Magnaporthe oryzae* using MapMan 3.6.0. ‘Wp+R vs. Wp’ (**a**) and ‘Cp+R vs. Cp’ (**b**) comparisons. Cp + R, *R. intraradices*-inoculated cultivated rice infected with *M. oryzae*; Cp, *R. intraradices*-uninoculated cultivated rice infected with *M. oryzae*; Wp + R, *R. intraradices*-inoculated wild rice infected with *M. oryzae*; Wp, *R. intraradices*-uninoculated wild rice infected with *M. oryzae*. Red color intensity indicates the levels of up-regulation by log_2_ (fold-change) > 0 and *P* value < 0.05. Blue color intensity indicates the levels of down-regulation by log_2_ (fold-change) < 0 and *P* value < 0.05

MapMan 3.6.0 software was used to identify biotic stress-related genes in the ‘Wp+R vs. Wp’ and ‘Cp+R vs. Cp’ comparisons*.* Results revealed that more signaling genes exhibited altered expression in the ‘Wp + R vs. Wp’ comparison than in the ‘Cp + R vs. Cp’ comparison (Fig. 6). Peroxidases encoding genes were significantly up-regulated in the ‘Wp + R vs. Wp’ comparison, while they were down-regulated in the ‘Cp + R vs. Cp’ comparison. In particular, two signaling genes (*Os05g0418100* and *Os11g0181400*) encoding the Mildew Resistance Locus O (MLO)-like proteins were induced in the ‘Wp + R vs. Wp’ comparison. The ß-glucanase-related genes *Os08g0244500* and *Os01g0713200* (encoding 1,3-ß-glucosidases), were also significantly up-regulated in the ‘Wp + R vs. Wp’ comparison, while *Os07g0539400* and *Os07g0600700*, encoding endo-1,3-ß-glucosidases, were up-regulated in the ‘Cp + R vs. Cp’ comparison (Table 5; Additional file 5: Table S4a). The cell wall-related genes *Os09g0422500*, *Os01g0750300* and *Os01g0746700* were up-regulated in the ‘Wp + R vs. Wp’ comparison, while *Os08g0345500*, *Os08g0160500* and *Os09g0428000*, encoding cellulose synthases, were up-regulated in the ‘Cp + R vs. Cp’ comparison (Fig. 6; Table 5; Additional file 5: Table S4b).

As for the genes related to JA and SA, the two hormones with well-known regulatory roles in plant responses to biotic stresses (Nasir et al. 2018; AbuQamar et al. 2017), the JA-related genes *Os02g0218700* and *Os03g0738600*, which encode allene oxide synthase and lipoxygenase, respectively, were up-regulated in the ‘Wp+R vs. Wp’ comparison (Fig. 6; Table 5; Additional file 5: Table S4a). On the other hand, four SA-related genes encoding carboxyl methyltransferases, of which *Os11g0256900* and *Os04g0665200* were up-regulated in the ‘Wp + R vs. Wp’ comparison, while *Os11g0260100* and *Os01g0701700* were up-regulated in the ‘Cp+R vs. Cp’ comparison (Fig. 6; Table 5; Additional file 5: Table S4b).

### Comparative analysis of changes in secondary metabolism

**Fig. 7:**
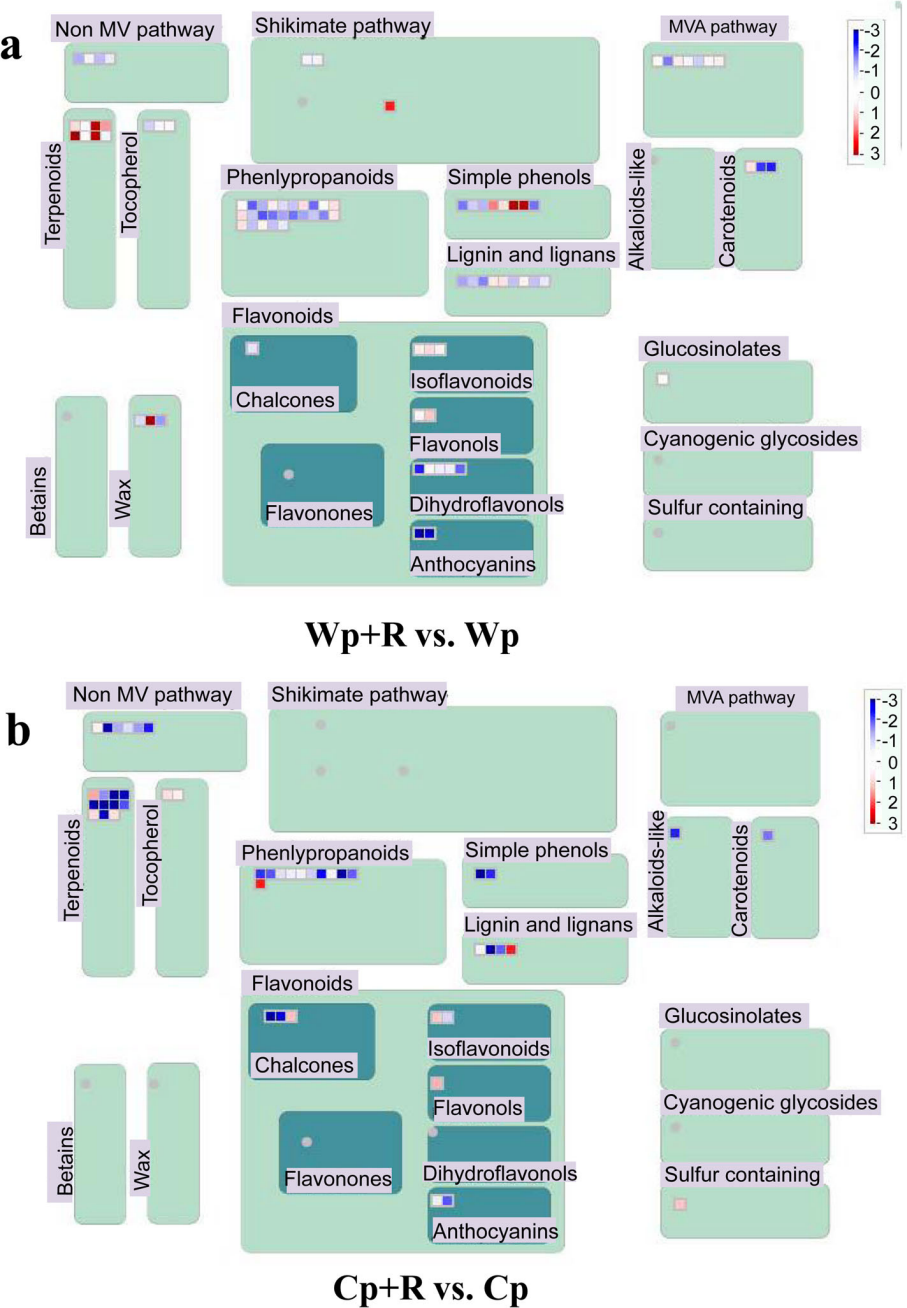
Analysis of secondary metabolic pathway of *Rhizoglomus intraradices*-inoculated plants to infection by *Magnaporthe oryzae* using MapMan 3.6.0. ‘Wp+R vs. Wp’ (**a**) and the ‘Cp+R vs. Cp’ (**b**) comparisons. Cp + R, *R. intraradices*-inoculated cultivated rice infected with *M. oryzae*; Cp, *R. intraradices*-uninoculated cultivated rice infected with *M. oryzae*; Wp + R, *R. intraradices*-inoculated wild rice infected with *M. oryzae*; Wp, *R. intraradices*-uninoculated wild rice infected with *M. oryzae*. Red color intensity indicates the DEGs with log_2_ (fold-change) > 0 and *P* value < 0.05, and blue color intensity indicates the DEGs with log_2_ (fold-change) < 0 and *P* value < 0.05 according to the scale bar

MapMan 3.6.0 software was also used to identify changes in secondary metabolism in the ‘Wp+R vs. Wp’ and ‘Cp+R vs. Cp’ comparisons with reference to the obtained DEGs. With respect to the shikimate pathway, results indicated that the gene *Os01g0375500*, encoding a shikimate 5-dehydrogenase, was significantly up-regulated in the ‘Wp + R vs. Wp’ comparison, whereas no shikimate pathway-genes with altered expression was detected in ‘Cp + R vs. Cp’ comparison (Fig. 7; Table 5; Additional file 6: Table S5a). In addition, seven mevalonate pathway (MVA)-related genes (e.g., *Os03g0118800* and *Os09g0492700*) were found to be differentially expressed (i.e. 4 up- and 3 down-regulated genes) in the ‘Wp + R vs. Wp’ comparison (Fig. 7; Table 5; Additional file 6: Table S5b), while no MVA-related DEGs were identified in ‘Cp + R vs. Cp’ comparison. A number of simple phenols-related genes (e.g., *Os07g0101000* and *Os05g0458300*) were up-regulated in the ‘Wp + R vs. Wp’ comparison, while only down-regulated phenols-related genes (e.g., *Os02g0749700* and *Os12g0258700*) were detected in the ‘Cp + R vs. Cp’ comparison (Fig. 7; Table 5; Additional file 6: Table S5c). As for the terpenoid pathway-related DEGs, a majority of analyzed DEGs, i.e. 7 of 8, showed up-regulated expression (e.g., *Os02g0570400* and *Os04g0344100*) in the ‘Wp + R vs. Wp’ comparison, whereas a higher number of the terpenoid pathway-related DEGs detected in the ‘Cp + R vs. Cp’ comparison (8 of 11) were down-regulated genes (e.g., *Os02g0568700* and *Os02g0571100*) (Fig. 7; Table 5; Additional file 6: Table S5d).

## Discussion

Mycorrhizal interactions are mutually beneficial; specifically, AMFs cannot only improve nutrient absorption in their host plants (e.g., *Lycopersicon esculentum* and *Triticum aestivum*) but also increase resistance of host plants to biotic (e.g., pathogen) and abiotic (e.g., drought and water) stresses (Chitarra et al. 2016; Brito et al. 2018; Fiorilli et al. 2018; Sánchez-Romera et al. 2018; Volpe et al. 2018). Pathogenic fungi can cause serious damage to crop production (Croll and McDonald 2017; Syed Ab Rahman et al. 2018). Since rice can form a mycorrhizal relationship with AMFs, it is important to understand what mutual benefits ensue from this interaction. The present study demonstrated that both wild and cultivated rice could be effectively colonized by the AMF *R. intraradices* (Fig. 2a and b), which could thereby provide strong protection against *M. oryzae* infection (Fig. 3). In addition, our data also indicated the abundance of *R. intraradices* was significantly higher in the wild rice rhizosphere than it was in the rhizosphere of cultivated rice (Fig. 2d). This result may indicate that wild rice can attract, host and sustain a greater level of AMFs in their rhizosphere than cultivated rice. This finding is consistent with results of a previous study, which showed that the relative abundance of AMFs was higher in wild rice than in cultivated rice (Shi et al. 2018a). In support of these findings, a recent study reported that the domestication of different crop species, including maize (*Zea mays*), barley (*Hordeum vulgare*) and wheat (*T. durum* Desf.), reduced the benefits obtained from AMFs (Martín-Robles et al. 2018).

AMFs can enhance the ability of a host plant to resist pathogenic fungi by inducing plant resistance systems (Cameron et al. 2013; Pérez-de-Luque et al. 2017). The phenotypes of *R. intraradices*-inoculated wild and cultivated rice plants infected with *M. oryzae* revealed that *R. intraradices* could improve the resistance of both wild and cultivated rice to the pathogen (Fig. 3), clearly demonstrating that AMF(s) can help plants resist pathogenic fungi. The results of the study also demonstrated, however, that AMF-inoculated wild rice were more resistant to *M. oryzae* than AMF-inoculated cultivated rice (Fig. 3), indicating that *R. intraradices* played a more substantial role in the resistance response to *M. oryzae* in wild rice than it did in cultivated rice which might be associated with its higher root colonization rate and abundance observed in wild rice versus cultivated rice (Fig. 3c and d). Thus, we hypothesize that the difference in the resistant responses of wild and cultivated rice to *M. oryzae* (or other pathogens or abiotic stresses) may partially be due to the increased beneficial effects of the AMFs that have been retained in wild rice and lost or reduced in domesticated cultivated rice.

Transcriptome data provide information on gene expression and are commonly used to identify functional differences in plant responses to biotic and abiotic stresses (Nasir et al. 2018; Abdelrahman et al. 2018). Our data identified a greater number of DEGs in ‘Wp+R vs. Wp’ comparison (*R. intraradices*-inoculated wild rice infected with *M. oryzae* vs. *R. intraradices*-uninoculated wild rice infected with *M. oryzae*) than in ‘Cp+R vs. Cp’ comparison (*R. intraradices*-inoculated cultivated rice vs. *R. intraradices*-uninoculated cultivated rice infected with *M. oryzae*) (Fig. 4). This may indicate that the resistance response in *R. intraradices*-inoculated wild rice is more complex than that in *R. intraradices*-inoculated cultivated rice. The KEGG enrichment analysis revealed that in comparison with *R. intraradices*-uninoculated control, both the *R. intraradices*-inoculated wild and cultivated rice contained up-regulated genes related to auxin signal transduction or biosynthesis, with more up-regulated genes being identified in ‘Wp + R vs. Wp’ comparison than ‘Cp + R vs. Cp’ comparison (12 vs. 8) (Additional file 4: Table S3). These data suggested that the AMF *R. intraradices* enhanced auxin signal transduction in both the wild and cultivated rice, and perhaps higher enhancement would occur in *R. intraradices*-inoculated wild than *R. intraradices*-inoculated cultivated rice during *M. oryzae* infection. Auxin is one of the plant growth regulators, which supports the establishment of mycorrhizal association between *R. irregularis* and rice (Etemadi et al. 2014; Pozo et al. 2015). In a good agreement, we also observed higher root colonization, as well as soil *R. intraradices* abundance, in wild than cultivated rice inoculated with *R. intraradices* and infected with *M. oryzae* (Fig. 2a-c). Interestingly, the KEGG results showed that ‘brassinosteroid biosynthesis’ was enriched in the ‘Cp + R vs. Cp’ comparison only, with 3 up-regulated brassinosteroids-related genes being found in ‘Cp + R vs. Cp’ comparison but none in ‘Wp + R vs. Wp’ comparison (Table 6). Brassinosteroids have been shown to function as disease-resistance-related hormones by inducing the production of reactive oxygen species (ROS), which might play important roles in disease resistance in plants as signaling molecules (Xia et al. 2009), suggesting that enhancement of brassinosteroids-related pathway is essential for survival of *R. intraradices*-inoculated cultivated rice during the invasion of *M. oryzae*.

JA and SA are plant hormones that have been known to play important roles in enhancing the resistance of plants particularly to various kinds of biotic stressors (Agrawal et al. 2003; Bonnet et al. 2017; do Prado Ribeiro et al. 2018). JA synthesis requires linolenic acid as a precursor (Iwanami et al. 2017), and JA can trigger ISR in plants that helps plants survive pathogenic conditions (Mehari et al. 2015; Zebelo et al. 2016). In our study, the JA-related genes *Os02g0218700* (encoding allene oxide synthase) and *Os03g0738600* (encoding lipoxygenase) were up-regulated in the ‘Wp+R vs. Wp’ comparison (Fig. 6; Additional file 5: Table S4c). Allene oxide synthase and lipoxygenase are key enzymes in the biosynthesis of JA, suggesting that *R. intraradices* might have activated the ISR in wild rice. SA can induce the synthesis of pathogenesis-related (PR) proteins in plants that play a role in plant defense against pathogenic fungi, and are directly involved in the plant immune system (Birkenbihl et al. 2017; Mahesh et al. 2017). The SA-related genes *Os11g0256900* and *Os04g0665200*, which encode carboxyl methyltransferases - the key enzymes in the biosynthesis of SA, were up-regulated in the ‘Wp + R vs. Wp’ comparison. On the other hand, *Os11g0260100* and *Os01g0701700,* which were also predicted to encode carboxyl methyltransferases, were up-regulated in the ‘Cp+R vs. Cp’ comparison (Fig. 6; Additional file 5: Table S4d). These findings together suggest that SA-related pathway might be activated in both rice genotypes by *R. intraradices* to enhance their resistance to *M. oryzae*.

ß-glucanases are proteins that can degrade the cell walls of pathogenic fungi, including *M. oryzae* (Samalova et al. 2017). In the present study, the ß-glucanase-related genes *Os08g0244500* and *OS01g0713200* (encoding 1,3-ß-glucosidases) were significantly up-regulated in the ‘Wp+R vs. Wp’ comparison, while *Os07g0539400* and *Os07g0600700*, encoding endo-1,3-ß-glucosidases, were up-regulated in the ‘Cp+R vs. Cp’ comparison (Fig. 6; Table 5; Additional file 5: Table S4a). Cellulose also plays an important role in cell wall structure and disease resistance (Kesten et al. 2017). The cellulose synthase-encoding genes *Os09g0422500*, *Os01g0750300* and *Os01g0746700* were up-regulated in the ‘Wp + R vs. Wp’ comparison, whereas other cellulose synthase-encoding genes like *Os08g0345500*, *Os08g0160500* and *Os09g0428000* were up-regulated in the ‘Cp + R vs. Cp’ comparison (Fig. 6; Table 5; Additional file 5: Table S4b). These data indicate that *R. intraradices* induced the plants to express more ß-glucanases and celluloses in both wild and cultivated rice in response to *M. oryzae* as a means to protect both varieties from the infection.

The transcriptome data indicated that the shikimate pathway-related gene *Os01g0375500*, encoding shikimate 5-dehydrogenase, was significantly up-regulated in the ‘Wp+R vs. Wp’ comparison but not in the ‘Cp+R vs. Cp’ comparison (Fig. 7a; Table 5; Additional file 6: Table S5a). The synthesis of the SA phytohormone proceeds through the shikimic acid pathway (Shine et al. 2016), suggesting the contribution of up-regulated shikimate pathway to enhancement of SA signaling. With respect to the MVA pathway that is the synthetic pathway for terpenoids, 4 up- and 3 down-regulated genes were identified in the ‘Wp + R vs. Wp’ but no DEGs were detected ‘Cp + R vs. Cp’ comparison (Fig. 7a; Additional file 6: Table S5b), providing evidence for the implication of the MVA pathway in response of *R. intraradices*-inoculated wild rice to *M. oryzae*. Additionally, simple phenols-related pathways might be better enhanced in *R. intraradices*-inoculated wild rice than in *R. intraradices*-inoculated cultivated rice after their infection with *M. oryzae*, as several up-regulated genes were recorded in the ‘Wp + R vs. Wp’ comparison, while only down-regulated genes were found in the ‘Cp + R vs. Cp’ comparison (Fig. 7). Similarly, a majority of the terpenoid pathway-related genes were up-regulated in the ‘Wp+R vs. Wp’, while down-regulated in ‘Cp + R vs. Cp’ comparison. These findings together might suggest the contribution of the enhanced shikimate, MVA, phenolic and terpenoid pathways to the higher resistance of wild rice versus that of cultivated rice to *M. oryzae* by the inoculation with *R. intraradices* (Fig. 7). In support of our results, increasing evidence has suggested that the shikimate, MVA, terpenoid and small phenols-related metabolic pathways play important roles in the ability of plants to resist fungal infection (Kuc 1995; Martinelli et al. 2015; Massalha et al. 2017; Martinez et al. 2018; Masi et al. 2018; Takemoto et al. 2018). For instance, shikimate pathway, which promotes the synthesis of phenylpropanoids and then the synthesis of SA, is involved in regulation of plant resistance to fungal pathogens (Shi et al. 2018b). Terpenoids and small phenols can serve as phytoalexins that can promote plant in resisting diseases (Meyer et al. 2017; Sun et al. 2017). AMFs can induce the MVA, which in turn may stimulate the biosynthesis of small phenols and terpenoids, thereby contributing to improving disease resistance (Fontana et al. 2009; Kapoor et al. 2017).

**Fig. 8:**
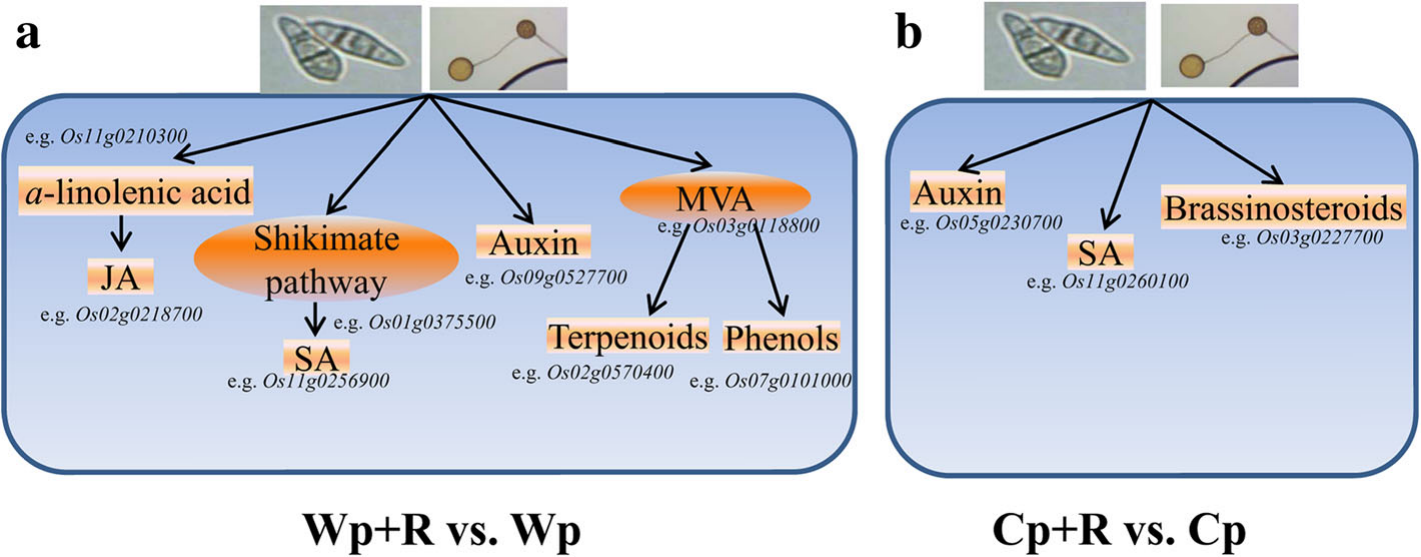
Diagrammatic model of the responses of *Rhizoglomus intraradices*-uninoculated and *R. intraradices*-inoculated wild (**a**) and cultivated (**b**) rice to *Magnaporthe oryzae* infection. Cp + R, *R. intraradices*-inoculated cultivated rice infected with *M. oryzae*; Cp, *R. intraradices*-uninoculated cultivated rice infected with *M. oryzae*; Wp + R, *R. intraradices*-inoculated wild rice infected with *M. oryzae*; Wp, *R. intraradices*-uninoculated wild rice infected with *M. oryzae*. SA, salicylic acid; JA, jasmonic acid; MVA, mevalonate pathway

As a result of our comparative analyses, we were able to construct a model of the different responses of *R. intraradices*-inoculated wild and cultivated rice to *M. oryzae* (Fig. 8). *R. intraradices* contributed to the regulation of auxin and SA metabolism in both wild and cultivated rice in response to *M. oryzae*. Furthermore, *R. intraradices* might increase α-linolenic acid production, thereby improving JA synthesis in wild rice; as well as induce the shikimate pathway, leading to alterations in downstream metabolism in wild rice. *R. intraradices* also induced the MVA pathway, and thus increased the syntheses of terpenoids and phenols in wild rice in response to *M. oryzae* (Fig. 8a). On the other hand, *R. intraradices* enhanced the biosynthesis of brassinosteroids in cultivated rice leading to enhanced plant resistance to the pathogenic *M. oryzae* (Fig. 8b).

## Conclusions

In this study, we reported evidence that the benefits received by host plants from mycorrhizal associations differed between cultivated and wild rice, which might have been altered during the process of rice domestication. Our data suggest that number or level of symbiotic benefits has decreased in the cultivated rice relative to wild rice, which may result in the activation of different disease responses when cultivated rice plants are challenged by a pathogenic fungus.

## Additional files

Additional file 1: **Table S1.** Genes and primers used for verifying RNA-sequencing data using RT-qPCR. (DOC 46 kb)

Additional file 2: **Figure S1.** Lesion areas of wild and cultivated rice plants in response to *Magnaporthe oryzae* infection with and without inoculation with the arbuscular mycorrhizal fungus *Rhizoglomus intraradices*. Cp + R, *R. intraradices*-inoculated cultivated rice infected with *M. oryzae*; Cp, *R. intraradices*-uninoculated cultivated rice infected with *M. oryzae*; Wp + R, *R. intraradices*-inoculated wild rice infected with *M. oryzae*; Wp, *R. intraradices*-uninoculated wild rice infected with *M. oryzae*. (TIF 141 kb)

Additional file 3 **Table S2.** RNA-sequencing and reverse transcription-quantitative real-time PCR (RT-qPCR) data of verified genes. The log_2_ (fold-changes) and fold-changes shown were obtained from RNA-sequencing and RT-qPCR data derived from the ‘Cp+R vs. Cp’ comparison and ‘Wp+R vs. Wp’ comparison, respectively. Red, blue and black colors indicate the up-regulated [log_2_(fold-change) > 0, *q*-value < 0.05 in RNA-sequencing data, or fold-change ≥2 in RT-qPCR data with a *P*-value < 0.05], down-regulated [log_2_(fold-change) < 0, *q*-value < 0.05 in RNA-sequencing data, or fold-change ≤2 in RT-qPCR data with a *P*-value < 0.05] and unchanged genes, respectively. Cp + R, *Rhizoglomus intraradices* -inoculated cultivated rice infected with *Magnaporthe oryzae*; Cp, *R. intraradices*-uninoculated cultivated rice infected with *M. oryzae*; Wp + R, *R. intraradices*-inoculated wild rice infected with *M. oryzae*; Wp, *R. intraradices*-uninoculated wild rice infected with *M. oryzae*. (DOCX 32 kb)

Additional file 4: **Table S3.** KEGG analysis of the response of *Rhizoglomus intraradices*-inoculated wild rice plants to infection by *Magnaporthe oryzae* in the ‘Wp+R vs. Wp’ (a) and ‘Cp+R vs. Cp’ (b) comparisons. Cp + R, AMF-inoculated cultivated rice infected with *M. oryzae*; Cp, AMF-uninoculated cultivated rice infected with *M. oryzae*; Wp + R, AMF-inoculated wild rice infected with *M. oryzae*; Wp, AMF-uninoculated wild rice infected with *M. oryzae*. (XLS 63 kb)

Additional file 5: **Table S4.** MapMan 3.6.0 analysis of biotic stress response for ß-glucanase (a), cell wall (b), JA (c) and SA (d) in AMF-inoculated wild and cultivated rice in response to infection by *Magnaporthe oryzae* in the ‘Wp+R vs. Wp’ and ‘Cp+R vs. Cp’ comparisons. Cp + R, AMF-inoculated cultivated rice infected with *M. oryzae*; Cp, AMF-uninoculated cultivated rice infected with *M. oryzae*; Wp + R, AMF-inoculated wild rice infected with *M. oryzae*; Wp, AMF-uninoculated wild rice infected with *M. oryzae*. SA, salicylic acid; JA, jasmonic acid. (XLS 93 kb)

Additional file 6 **Table S5.** MapMan 3.6.0 analysis of secondary metabolic pathway response for shikimate pathway (a), MVA (b), simple phenols (c) and terpenoids (d) in AMF-inoculated wild and cultivated rice in response to infection by *Magnaporthe oryzae* in the ‘Wp+R vs. Wp’ and ‘Cp+R vs. Cp’ comparisons. Cp + R, AMF-inoculated cultivated rice infected with *M. oryzae*; Cp, AMF-uninoculated cultivated rice infected with *M. oryzae*; Wp + R, AMF-inoculated wild rice infected with *M. oryzae*; Wp, AMF-uninoculated wild rice infected with *M. oryzae*. MVA, mevalonate. (XLS 46 kb)
