# Supplementary material for: Comparative study of the mycorrhizal root transcriptomes of wild and cultivated rice in response to the pathogen Magnaporthe oryzae
Source: Rice (N Y). 2019 May 10;12:35. doi: 10.1186/s12284-019-0287-9 (PMC6510786; doi:10.1186/s12284-019-0287-9)
Supplement: Supplementary file 1 — Table S1. Genes and primers used for verifying RNA-sequencing data using RT-qPCR. (DOC 46 kb) [file 12284_2019_287_MOESM1_ESM.doc]

**Additional file 1: Table S1** Genes and primers used for verifying RNA-sequencing data using RT-qPCR.

| Gene ID | Primer name | Sequence (5'->3') |
| --- | --- | --- |
| *Os12g0168700* | OS12G0168700-F | TCTGCACTCAAGCCAACACT |
|  | OS12G0168700-R | CCAACTTCCATTGACTGCGG |
| *Os06g0726200* | OS06G0726200-F | CCGACCGGATTGGGTTCTAC |
|  | OS06G0726200-R | AGCCATTGTGGGCATTACTGA |
| *Os04g0229100* | OS04G0229100-F | CCAGAAGCAGATGCAGGCTA |
|  | OS04G0229100-R | ACTCACCGTCCTCTTACCGA |
| *Os02g0627100* | OS02G0627100-F | GTATCCGCTCTACCGGTTCG |
|  | OS02G0627100-R | GCCTCCACACTCCACTGTTAT |
| *Os01g0854800* | OS01G0854800-F | GAGATCGCCTCCGTTCTCAG |
|  | OS01G0854800-R | ACGTACTTGGAATCCTGCGG |
| *Os01g0892500* | OS01G0892500-F | TCATGCACGATTTCCTAAGGA |
|  | OS01G0892500-R | GCGGTGATGCTCTTGACAAG |
| *Os02g0175000* | OS02G0175000-F | CCCAGGGCACAAAAGTTACA |
|  | OS02G0175000-R | ACGATGATCCCAATGCCTTT |
| *Os02g0678200* | OS02G0678200-F | TGGAGGAGTGGAAAGAGTACT |
|  | OS02G0678200-R | TCAATCTGATCATCAAGCATCCT |
| *β-Tubulin* | Tubulin-F | TACCGTGCCCTTACTGTTCC |
|  | Tubulin-R | CGGTGGAATGTCACAGACAC |
