# Supplementary figures and images for: Comparative study of the mycorrhizal root transcriptomes of wild and cultivated rice in response to the pathogen Magnaporthe oryzae
Source: Rice (N Y). 2019 May 10;12:35. doi: 10.1186/s12284-019-0287-9 (PMC6510786; doi:10.1186/s12284-019-0287-9)

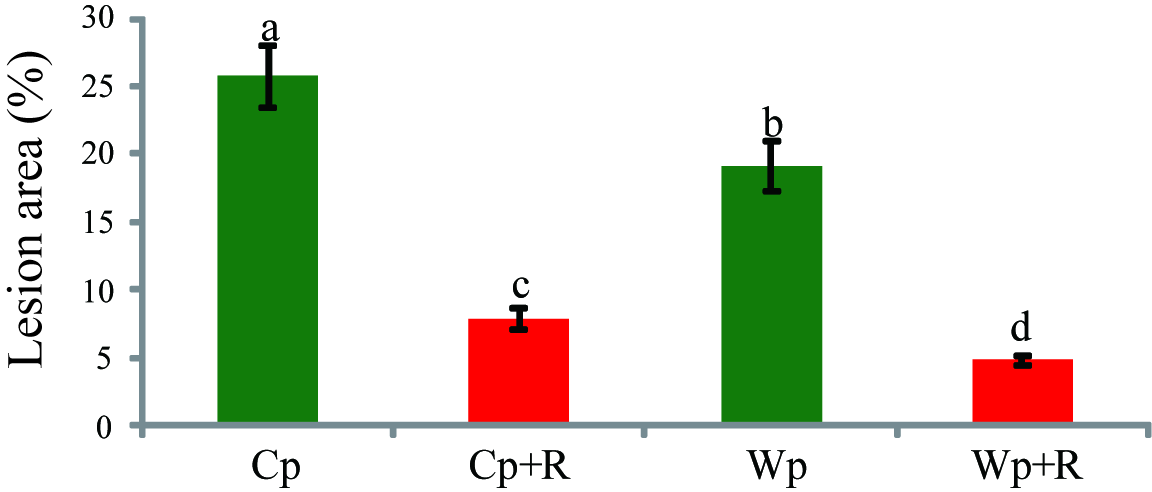

Supplement: Supplementary file 2 — Figure S1. Lesion areas of wild and cultivated rice plants in response to Magnaporthe oryzae infection with and without inoculation with the arbuscular mycorrhizal fungus Rhizoglomus intraradices. Cp + R, R. intraradices-inoculated cultivated rice infected with M. oryzae; Cp, R. intraradices-uninoculated cultivated rice infected with M. oryzae; Wp + R, R. intraradices-inoculated wild rice infected with M. oryzae; Wp, R. intraradices-uninoculated wild rice infected with M. oryzae. (TIF 141 kb) [file 12284_2019_287_MOESM2_ESM.tif]
