# Supplementary material for: Comparative study of the mycorrhizal root transcriptomes of wild and cultivated rice in response to the pathogen Magnaporthe oryzae
Source: Rice (N Y). 2019 May 10;12:35. doi: 10.1186/s12284-019-0287-9 (PMC6510786; doi:10.1186/s12284-019-0287-9)
Supplement: Supplementary file 3 — Table S2. RNA-sequencing and reverse transcription-quantitative real-time PCR (RT-qPCR) data of verified genes. The log2 (fold-changes) and fold-changes shown were obtained from RNA-sequencing and RT-qPCR data derived from the ‘Cp+R vs. Cp’ comparison and ‘Wp+R vs. Wp’ comparison, respectively. Red, blue and black colors indicate the up-regulated [log2(fold-change) > 0, q-value < 0.05 in RNA-sequencing data, or fold-change ≥2 in RT-qPCR data with a P-value < 0.05], down-regulated [log2(fold-change) < 0, q-value < 0.05 in RNA-sequencing data, or fold-change ≤2 in RT-qPCR data with a P-value < 0.05] and unchanged genes, respectively. Cp + R, Rhizoglomus intraradices -inoculated cultivated rice infected with Magnaporthe oryzae; Cp, R. intraradices-uninoculated cultivated rice infected with M. oryzae; Wp + R, R. intraradices-inoculated wild rice infected with M. oryzae; Wp, R. intraradices-uninoculated wild rice infected with M. oryzae. (DOCX 32 kb) [file 12284_2019_287_MOESM3_ESM.docx]

**Additional file 3: Table S2** RNA-sequencing and reverse transcription-quantitative real-time PCR (RT-qPCR) data of verified genes. The log_2_ (fold-changes) and fold-changes shown were obtained from RNA-sequencing and RT-qPCR data derived from the ‘Cp+R vs. Cp’ comparison and ‘Wp+R vs. Wp’ comparison, respectively. Red, blue and black colors indicate the up-regulated [log_2_(fold-change)>0, *q*-value < 0.05 in RNA-sequencing data, or fold-change ≥2 in RT-qPCR data with a *P*-value <0.05], down-regulated [log_2_(fold-change) <0, *q*-value < 0.05 in RNA-sequencing data, or fold-change ≤2 in RT-qPCR data with a *P*-value <0.05] and unchanged genes, respectively. Cp+R, *Rhizoglomus intraradices* -inoculated cultivated rice infected with *Magnaporthe oryzae*; Cp, *R. intraradices*-uninoculated cultivated rice infected with *M. oryzae*; Wp+R, *R. intraradices*-inoculated wild rice infected with *M. oryzae*, Wp, *R. intraradices*-uninoculated wild rice infected with *M. oryzae*.

| Gene ID | RNA-sequencing data | | | | RT-qPCR data | | | |
| --- | --- | --- | --- | --- | --- | --- | --- | --- |
|  | Cp+R vs. Cp | | Wp+R vs. Wp | | Cp+R vs. Cp | | Wp+R vs. Wp | |
|  | Log_2_ (fold-change) | *q*-value | Log_2_ (fold-change) | *q*-value | Fold-change | *P*-value | Fold-change | *P*-value |
| *Os12g0168700* | No change | >0.05 | -0.77165 | <0.01 | 1.58 | 0.16 | -2.13 | <0.01 |
| *Os06g0726200* | No change | >0.05 | -0.83337 | <0.01 | -1.07 | 0.21 | -2.03 | <0.01 |
| *Os04g0229100* | No change | >0.05 | -1.1788 | <0.01 | 1.71 | 0.09 | -2.46 | <0.01 |
| *Os02g0627100* | No change | >0.05 | -1.6726 | <0.01 | 1.35 | 0.07 | -2.11 | <0.01 |
| *Os01g0854800* | No change | >0.05 | 1.9953 | <0.01 | -1.04 | 0.21 | 2.48 | <0.01 |
| *Os01g0892500* | 3.1927 | <0.01 | No change | >0.05 | 2.42 | <0.01 | -1.66 | 0.09 |
| *Os02g0175000* | 2.6359 | <0.01 | No change | >0.05 | 9.41 | <0.01 | 1.10 | 0.13 |
| *Os02g0678200* | 2.0198 | <0.01 | No change | >0.05 | 12.36 | <0.01 | -1.56 | 0.23 |
